# Subetta Treatment Increases Adiponectin Secretion by Mature Human Adipocytes *In Vitro*

**DOI:** 10.1155/2013/925874

**Published:** 2013-04-18

**Authors:** Jim Nicoll, Evgeniy A. Gorbunov, Sergey A. Tarasov, Oleg I. Epstein

**Affiliations:** ^1^Zen-Bio, Inc., 3200 Chapel Hill-Nelson Boulevard, Street 100, Research Triangle Park, NC 27709, USA; ^2^OOO “NPF “MATERIA MEDICA HOLDING”, 3rd Samotyochny per., 9, Moscow 127473, Russia

## Abstract

*Purpose*. To investigate the mechanism of action in peripheral tissues of novel complex drug containing release-active dilutions of antibodies to the beta subunit of the insulin receptor and antibodies to endothelial nitric oxide synthase (Subetta), which has shown efficacy in animal models of diabetes. *Methods*. Human mature adipocytes were incubated either with Subetta, with one of negative controls (placebo or vehicle), with one of nonspecific controls (release-active dilutions of antibodies to cannabinoid receptor type I or release-active dilutions of rabbit nonimmune serum), or with dimethyl sulfoxide (DMSO) at 37°C in a humidified incubator at 5% CO_2_ for three days. Rosiglitazone was used as reference drug. Secretion of adiponectin was measured by quantitative enzyme-linked immunosorbent assay (ELISA). *Results*. Only Subetta significantly stimulates adiponectin production by mature human adipocytes. Nonspecific controls did not significantly affect adiponectin secretion, resulting in adiponectin levels comparable to background values of the negative controls and DMSO. *Conclusion*. Increasing adiponectin production in absence of insulin by Subetta probably via modulating effect on the beta subunit of the insulin receptor might serve as one of the mechanisms of the antidiabetic effect of this drug. These *in vitro* results give first insight on possible mechanism of action of Subetta and serve as a background for further studies.

## 1. Introduction

According to World Health Organization (WHO) (2012) more than 347 million people worldwide suffer from diabetes mellitus (DM), of which 90% have type 2 diabetes (T2D) [1]. Both the prevalence and morbidity, especially of T2D, continue to grow globally, especially in developing countries. This growth has led to strains on healthcare systems worldwide as T2D remains one of the leading causes of cardiovascular disorders, blindness, renal failure, amputations, and hospitalizations [2].

Adiponectin is a circulating protein produced by adipose tissue that is a versatile regulator of energy homeostasis, insulin sensitisation, inflammation/atherosclerotic processes, and anti-ischemic cardioprotection [3]. It is well established that adiponectin plays an important role in T2D, hypertension, multiple sclerosis, and dyslipidaemias [4]. Particularly, there is a strong association between hypoadiponectinemia and T2D [3]: both adipose adiponectin mRNA expression and circulating adiponectin levels are significantly reduced in most rodent models of T2D, as well as T2D patients; the degree of glycosylated adiponectin and “high-molecular weight/total adiponectin ratio,” which correlates with insulin sensitivity, were significantly decreased in T2D patients compared to healthy controls; high adiponectin levels are associated with reduced risk of developing diabetes.

The most significant role adiponectin may play is that of sensitizing the liver and muscles to the action of insulin in both humans and rodents [5]. Adiponectin appears to increase insulin sensitivity by improving glucose and lipid metabolism. Adiponectin has an ameliorating function on glucose metabolism apart from insulin signalling [6]. In animal models of obesity and diabetes, adiponectin affected both skeletal muscle and liver, promoting fatty acid oxidation in muscle and inhibiting glucose production from the liver, thereby leading to decreases in circulating free fatty acids (FFAs), triglyceride, and glucose levels [7, 8]. FFAs as well as tumor necrosis factor alpha (TNF*α*) are factors released from adipose tissue that contribute to insulin resistance. In addition, lipid accumulation in beta-cells might lead to reductions in insulin secretion [7]. In individuals with visceral obesity, there is a decrease in the insulin-mediated suppression of lipolysis, leading to increases in circulating FFAs concentrations that contribute to both peripheral and hepatic insulin resistance either by impairing insulin signalling pathways, by competitive inhibition of muscle glucose uptake, by stimulation of endogenous glucose production, thus contributing to hepatic insulin resistance, or by combination of mentioned mechanisms [6, 7, 9, 10]. 

Also it was shown that adiponectin regulates the expression of several pro- and anti-inflammatory cytokines. Its main anti-inflammatory function might be related to its capacity to suppress the synthesis of TNF*α* and interferon gamma (IFNg) and to induce the production of anti-inflammatory cytokines such as interleukin-10 (IL-10) and IL-1 receptor antagonist (IL-1RA) [11]. TNF*α* is a key modulator of adipocyte metabolism, with a direct role in several insulin-mediated processes, including glucose homeostasis and lipid metabolism [6, 7, 12]. The net effect of TNF*α* is to decrease lipogenesis (FFA uptake and triglyceride synthesis) and to increase lipolysis. TNF*α* is a major contributor to the development of adipose tissue insulin resistance [7]. Finally, there is substantial evidence of adiponectin effects on beta-cell function and survival, which are well known as key factors in the development of T2D along with insulin resistance [12]. Taking into consideration the above mentioned it could be conclude that adiponectin-targeted pharmaceutical strategies increasing circulating adiponectin levels may be therapeutic against T2D.

Subetta is drug containing release-active dilutions of antibodies to the beta subunit of the insulin receptor and antibodies to endothelial nitric oxide (NO) synthase. This novel class of drug was demonstrated to have a fundamentally new proantigen (cotargeted with antigen) targeted activity [13]. For the first time in the study of the repeated dilution process, it was found that the resulting ultra-dilution generates a novel activity absent in the starting material, called a release-activity. A distinctive feature of the release-active antibodies is their ability not to suppress, and to modify the activity of the antigen against which they were raised.

In two DM animal models, streptozotocin-induced DM [14] and spontaneous T2D in Goto Kakizaki rats [15], Subetta showed antidiabetic effects similar to that of rosiglitazone. Additionally, Subetta has shown promising results in its first clinical use in both type 1 diabetes (T1D) patients with poor glycemic control receiving intensive insulinotherapy and T2D patients receiving metformin/combination of basal insulin with metformin (unpublished data). Subetta treatment led to a significant decrease in fasting plasma glucose and glycated haemoglobin (HbA_1C_) levels, exerted a normalizing effect on the daily glycemic levels (self-control of blood glucose and daily glucose monitoring), significantly lowered insulin resistance and plasma insulin levels, as well as lowering total cholesterol and low-density lipoproteins.

The aim of this work is to investigate the ability of Subetta to affect adiponectin secretion by mature human adipocytes.

## 2. Materials and Methods

Human preadipocytes (lot SL0047) were provided by Zen-Bio, Inc. (USA). Human preadipocytes were mixture of subcutaneous depots (abdomen, thigh, hip, flank, and breast) obtained from 5 healthy donors (sex: female; age: 47.0 ± 5.4) undergoing elective surgery that have signed informed consent, under existing institutional review board (IRB). The cells were pooled and grown together, then harvested and cryopreserved as the superlot (multidonor) that was used in the current study. Prior to use the cells undergo quality control in a number of functional assays (differentiation (measured by total triglyceride), lipolysis, insulin-induced glucose uptake) and also the results of the quality control have shown that there were not any differences between the depots. Preadipocyte medium (catalog number PM-1), adipocyte differentiation medium (catalog number DM-2), and adipocyte maintenance medium (catalog number AM-1) were provided by Zen-Bio, Inc. (USA). Enzyme-linked immunosorbent assay (ELISA) kit (catalog number ADIP-1) was the product of Zen-Bio, Inc. (USA). Rosiglitazone (catalog number R2408) was purchased from Sigma Aldrich (USA). Subetta, release-active dilutions (RAD) of antibodies (Abs) to the beta subunit of insulin receptor and Abs to endothelial NO synthase, RAD of Abs to cannabinoid receptor type I (R-CBI), RAD of rabbit nonimmune serum (RbS), RAD of purified water (placebo), and purified water were manufactured and supplied by OOO “NPF “MATERIA MEDICA HOLDING” (Russia). All of OOO “NPF “MATERIA MEDICA HOLDING” compounds, except for purified water, were manufactured using the method described previously [13] using routine methods described in the European Pharmacopoeia (6th Edition, 2007). All ultrahigh dilutions were prepared in glass vials. Starting substances were mixed with a solvent (ethanol-water solution) and shaken for 1 min to produce C1 dilution. All subsequent dilutions consisted of one part of the previous dilution to 99 parts of solvent (ethanol-water solution for intermediate dilutions and distilled water for preparation of the final dilution), with succession between each dilution. Solutions were prepared in sterile conditions, avoiding direct intense light, and were stored at room temperature. Rabbit polyclonal antibodies to the beta subunit of insulin receptor and to endothelial NO synthase were used as starting substances for Subetta, rabbit polyclonal antibodies to cannabinoid receptor type I—for RAD of Abs to R-CBI, rabbit nonimmune serum—for RAD of RbS. In case of placebo, purified water was used to prepare ultrahigh dilutions instead of starting substance.

The study was carried out by contract research organization Zen-Bio, Inc. (USA) in cooperation with OOO “NPF “MATERIA MEDICA HOLDING” (Russia). Human preadipocytes were plated at 40625 cells/cm^2^ in 150 *μ*L of preadipocyte medium. The cells were allowed to attach overnight in a 37°C humidified incubator at 5% CO_2_. The following day, the plating medium was removed and replaced with 150 *μ*L of adipocyte differentiation medium and the cells allowed to incubate in a 37°C humidified incubator at 5% CO_2_ for 1 week. After 1 week, 90 *μ*L of the differentiation medium was removed from each well and 120 *μ*L of adipocyte maintenance medium was added. The cells were then allowed to incubate in a 37°C humidified incubator at 5% CO_2_ for 7 more days. After this additional week, mature adipocytes were ready for assay.

Prior to the assay, the cells were incubated in the absence of serum and hormones for three days. After starvation, test compounds (75 *μ*L) were added and the cells were incubated at 37°C in a humidified incubator at 5% CO_2_ for three days. Conditioned media was collected after 72 hours and either frozen for later evaluation or measured immediately. For the ELISA, 20 *μ*L of the conditioned medium was added to 80 *μ*L of pretreatment solution and heated to 100°C for 5 minutes. Once the samples were cooled to room temperature, 50 *μ*L of each sample was added to 200 *μ*L diluent buffer to generate a 5-fold dilution. Samples were mixed and 100 *μ*L was transferred to the coated wells of the ELISA plate. Samples were incubated at room temperature for 1 hour after which the plate was washed and secondary antibody applied and incubated at room temperature for 1 hour. The plate was washed again and detection antibody was added and allowed to incubate for 1 hour at room temperature. Detection reagents were added and absorbance at 450 nm (SpectraMax 250: Molecular Devices) was determined.

The data are presented as mean value per group (M) ± standard deviation (SD). One-way analysis of variance (ANOVA) followed by Tukey HSD test was used for statistical analysis and *P* values less than 0.05 were regarded as significant (STATISTICA 6.1 software).

## 3. Results and Discussion

Incubation of mature human adipocytes with Subetta for 72 hours resulted in a statistically significant increase in adiponectin concentration in the culture medium (Table 1). Nonspecific controls, RAD of Abs to R-CBI and RAD of RbS, did not significantly affect adiponectin secretion, resulting in adiponectin levels comparable to background values of the negative controls, placebo, purified water, and 0.1% dimethyl sulfoxide (DMSO). The reference drug rosiglitazone stimulated adiponectin secretion but to a lower level compared to that of Subetta and its effect was not significant in comparison with DMSO value.

Previously, Subetta demonstrated the ability to reduce hyperglycemia and improve glucose tolerance in various experimental models of diabetes mellitus and its effects were similar to that of rosiglitazone [14, 15]. Subetta was given by oral gavage, when it was studied *in vivo* but in the current study the direct action of Subetta on mature human adipocytes was estimated *in vitro*. Based on the activity demonstrated for the whole class of novel drugs [13] and on data from previous studies, we may conclude that the complex drug mainly exerts a modulating effect on the beta subunit of the insulin receptor regulating the insulin receptor's kinase activity and consequently activating receptor-associated signal pathways [16]. The ability of direct activation of insulin receptor and activate receptor-associated signal pathways in the absence of insulin was shown for L7 (Merck) [17, 18].

According to Shehzad et al. [4], insulin directly stimulates adiponectin gene expression. Specifically, insulin enhances adiponectin regulation and secretion selectively in adipocytes. While the exact mechanism of action of insulin on adiponectin biosynthesis remains unclear, there are several possible pathways to account for its activity. It has been suggested that insulin binding to its receptor activates a cell cascade inhibiting the activity of FoxO1, a suppressor of peroxisome proliferator-activated receptor gamma (PPAR gamma), which in turn results in the induction of adiponectin biosynthesis [4]. It is important that adiponectin has an ameliorating function on glucose metabolism apart from insulin signalling [6].

Taking into consideration the above mentioned, it could be assumed that Subetta via direct effect on beta subunit of the insulin receptor of mature human adipocytes activates insulin receptor in the absence of insulin in culture medium, which in its turn exerts activating receptor-associated signal pathways. As a result activity of FoxO1 is inhibited and thus adiponectin biosynthesis is inducted. To confirm this suggestion, further studies (both *in vitro* and *in vivo*) should be conducted in order to provide a detailed investigation of the mechanism of Subetta influence on the production of adiponectin and also to assess the involvement of this process and its role in the mechanism of drug action.

## 4. Conclusions

In summary, the novel complex drug Subetta, containing RAD of antibodies to the beta subunit of insulin receptor and antibodies to endothelial NO synthase, stimulates adiponectin production by mature human adipocytes. This effect on adiponectin production is specific; neither RAD of Abs to R-CBI nor RAD of RbS affected adiponectin secretion by mature human adipocytes. Therefore, the ability of Subetta to exert its modulating effect on the insulin receptor even in absence of insulin might be the basis for stimulating adiponectin secretion and might serve as one of the mechanisms of the antidiabetic effect of this drug. The results of the current *in vitro* study give first insight on possible mechanism of action of Subetta and serve as a background for further studies.

## Figures and Tables

**Table 1:** Effect of Subetta and rosiglitazone on human adipocyte adiponectin secretion.

Sample	Adiponectin concentration (ng/mL)	*P* values (Subetta versus rest of the samples)	*P* values (Rosiglitazone versus rest of the samples)	Number of replicas
Subetta	27.97 ± 7.54	—	***P* = 0.028657**	6
RAD of Abs to R-CBI	4.66 ± 1.40	***P *** = 0.000134	*P* = 0.878582	5
RAD of RbS	5.18 ± 1.42	***P *** = 0.000123	*P* = 0.433495	6
Placebo	5.52 ± 0.69	***P *** = 0.000123	*P* = 0.491881	6
Purified water	8.73 ± 3.17	***P *** = 0.000422	*P* = 0.972188	6
DMSO (0.10%)	4.44 ± 1.27	***P *** = 0.000126	*P* = 0.452867	4
Rosiglitazone (1 *µ*M)	14.22 ± 2.94	***P *** = 0.028657	—	4

One-way analysis of variance (ANOVA) followed by Tukey HSD test was used for statistical analysis. ANOVA shows the following results: *F*_11/51_ = 16.4840, *P* = 0.00000, observed power = 1.0.
