# Expression of Concern on “Subetta Treatment Increases Adiponectin Secretion by Mature Human Adipocytes In Vitro”

**DOI:** 10.1155/2019/6595878

**Published:** 2019-06-25

**Authors:** International Journal of Endocrinology

*International Journal of Endocrinology* would like to express concern with the article titled “Subetta Treatment Increases Adiponectin Secretion by Mature Human Adipocytes In Vitro” [1]. After issues were raised by several different parties [2–4] and we consulted editors, concerns were found with conflicts of interest, the nature of the therapy as related to homeopathy, and the methods and reporting. Details are as follows.

## 1. Conflicts of Interest

Subetta (in Russian, Субетта), specifically “release-active dilutions of antibodies to *β*-subunit insulin receptor (RAD of Abs to *β*-InsR) and to endothelial nitric oxide synthase (RAD of Abs to eNOS)”, is a proprietary remedy owned by a company several of the authors are affiliated to, OOO “NPF” Materia Medica Holding; Sergey Tarasov is the Head of Research & Analytical Department, Oleg Epstein (also known as Epshtein) is the founder and CEO and holds related patents [5, 6], and Evgeniy Gorbunov is also affiliated. Though affiliations to and funding from OOO “NPF” Materia Medica Holding were stated in the article, this was not declared as a conflict of interest.

## 2. Homeopathy

The nature of Subetta as related to homeopathy should have been made clear. The serial dilutions used, C12, C30, and C200, make it likely that none of the original substance was left in the preparations. At C12, one molecule might remain; below this, none are likely to remain.

Dr. Epstein and colleagues have published on this topic since the late 1990s, first referring to “ultra-dilute potentized antibodies” and later coining the term “release-active dilutions” (RAD). The authors say RAD is not homeopathy [7], and some particles may be retained even at ultradilutions [8]; however, the description of the preparation is clearly related to homeopathy and RAD have been previously reported as related to homeopathy by this group: for example, a 2006 article stated that “ultralow concentrations of the antibody were obtained using routine homeopathic methods” and referred to their previous work as “drugs obtained by homeopathic methods” [9]; the patents note that the treatment is “prepared according to homeopathic technology” [5] and “homeopathically potentized” [6]; and a 2013 article by Dr. Epstein discusses homeopathy as a form of “release activity” and notes that production of RAD uses “Hahnemann's method” [10], i.e., homeopathic preparation, named after the founder of homeopathy, Samuel Hahnemann. Despite concerns over the plausibility of ultradilute antibodies having activity, in 2016 the United Kingdom High Court overturned a decision of the hearing officer of the Intellectual Property Office to refuse patents to Dr. Epstein's products [11].

## 3. Methods and Reporting

Subetta was reported to significantly increase adiponectin secretion compared to the positive control, a drug formerly used in diabetes treatment called rosiglitazone. However, rosiglitazone was reported to not significantly increase adiponectin secretion when compared to purified water, placebo, or DMSO (*p* values in the fourth column of Table 1). This would mean that the intended positive control did not work, but rerunning ANOVA with the Tukey HSD test shows that there are indeed statistically significant differences between rosiglitazone and the other controls (*p* < 0.05).

Samples of Subetta were stored at room temperature, but “storage at room temperature often leads to antibody degradation and/or inactivity, usually resulting from microbial growth” [12]. If the action of Subetta were dependent on antibody activity following ultradilution, storage at room temperature would have affected the results.

Some methodological details were missing. The catalogue number for the human preadipocytes from Zen-Bio is SP-F-SL. The glass vials were from Glastechnik Grafenroda Gmbh, Germany, were not coated, and were sterilized before use. There is no dose-response curve showing the effect of RAD at different dilutions.
